# Biology needs cyberinfrastructure to facilitate specimen-level data acquisition for insects and other hyperdiverse groups

**DOI:** 10.3897/zookeys.147.1944

**Published:** 2011-11-16

**Authors:** Wendy Moore

**Affiliations:** 1 Department of Entomology, University of Arizona, Tucson, Arizona 85721-0036, USA

The Internet has revolutionized the field of systematics by allowing for large scale cyberinfrastructure projects that (1) facilitate our work, such as ScratchPads (Smith et al. 2009), (2) provide outlets for the results of systematic work, such as the Tree of Life Web Project (Maddison et al. 2007), the Encyclopedia of Life (Wilson 2003), GenBank, MorphBank, MorphoBank, and (3) provide novel ways of online publishing (Blagoderov et al. 2010, Penev 2008). These efforts have greatly enhanced the field of systematics and are therefore enormously beneficial for understanding global biodiversity. However, specimen-level data are noticeably absent from most large-scale biodiversity cyberinfrastructure projects.

This situation is rapidly changing. For example, the NSF-sponsored “National Digital Biological Collections Resource” aims to digitize specimens held in natural history collections throughout North America in the next 10 years. This initiative will provide support for curatorial staff to digitize specimen-level data associated with their holdings. Meeting this goal for megadiverse groups, such as insects, will come with unique challenges. As a general rule, many specimens in most insect collections are not identified to the species level, while many are only identified to the family level.

For diverse families of insects, like carabid beetles with well over 35,000 described species, most experts are capable of making species level identifications of only a fraction of this diversity. In general they are too time-limited to meet this demand for large-scale ecological projects and initiatives such as the National Digital Biological Collections Resource (Anonymous 2000, Hopkins and Freckleton 2002). Not only is species identification an activity for which systematists receive little or no professional credit (McDade et al. 2011), it is also an activity for which the field has received criticism for not being able to provide this service to end users in a timely fashion. In the face of the biodiversity crisis, global climate change, and dwindling numbers of professional systematists, there is an overwhelming need to facilitate the acquisition of validated specimen-level data for biological research and science-informed conservation planning, and to disseminate these data broadly. In fact, the imbalance between limited expertise and the great need for expert identifications has led to efforts that attempt to meet these goals *without* the aid of expert systematists, such as the use of parataxonomists to identify morpho-species or operational taxonomic units (OTUs) or recognizable taxonomic units (RTUs) (Oliver and Beattie 1993, 1996), and the Barcode of Life project which is building a database of sequence data with the goal of allowing users to quickly and easily identify species molecularly in the future (Stockle and Hebert 2008). While both initiatives have their strengths, they have both been strongly criticized and both have been shown to fail in many instances (Barcode of Life: Will et al. 2005, Ebach 2011, Song et al. 2008; parataxonomists identifying morphospecies: Krell 2004).

The Internet provides an opportunity to develop creative solutions that do not sidestep, but enhance, the field of systematics. In order to accelerate the pace at which we can capture and disseminate validated specimen-level data for megadiverse groups such as insects, we need new cyberinfrastructure tools designed specifically for this purpose. Ideally these tools would be designed in such a way that they could easily be adopted by any interested working group of systematists and enhance the field of systematics.

While there are few cyberinfrastructure projects that focus at the specimen-level in entomology, AntWeb (www.antweb.org) is a notable exception and it serves as an example of the great potential of such a system. AntWeb is an online database that treats all species of ants worldwide focusing on specimen-level data and images contributed by a team of remote “curators.” AntWeb provides specimen-level data on ants to the Global Biodiversity Information Facility (GBIF) that, in turn, provides open access to biodiversity data.

Here, I describe a concept for such a system which would simultaneously add validated specimen-level data to existing cyberinfrastructure projects, track the contributions of data contributors and editors, and help to train the next generation of systematists. It would also promote the “publication” of specimen-level data as systematists acquire them. It would function much like AntWeb but it would allow working groups of systematists to adopt a pre-built, yet modifiable, cyberinfrastructure system and tailor it to their study group. For carabidologists, the time to move forward with a cyber-infrastructure solution could not be better. The National Ecological Observation Network (NEON) has chosen to monitor carabid beetles at 60 sites (20 core sites and 40 relocatable sites) throughout North America over the next 30 years. Therefore our field will soon be flooded with specimen-level data and requests for species identifications. There are not nearly enough carabidologists to perform the needed identifications of these specimens, especially when this activity is not a gold standard for promotion in most of the jobs we hold.

## Vision: Integration of Existing Components

The system would uniquely combine three existing, or relatively easy to develop, components, including (1) a password-protected working space for collections researchers, curatorial staff, and students, to interactively work with experts on specimen identifications, (2) a specimen-level database repository to store validated specimen records which would be linked to species-level web projects, and (3) taxon-specific products (see below).

1. A password-protected working space would consist of Internet portals that connect project **participants** (i.e., collections researchers, curatorial staff, and students) with project **editors** (specialists) in a virtual environment, including easy-to-use data entry forms and a secure environment for communication and training.

Data entry forms would include *at least* a photograph of the specimen, a tentative identification, georeferenced collecting information, *a unique specimen identifier* and the specimen repository. However the forms could also be much more elaborate and include any additional information that could be uniquely tied to a specimen, such as unique ecological data associated with the specimens, GenBank numbers, and BLAST results for genes extracted from the specimens.

Completed data entry forms would then be submitted through the system to the appropriate data editor who would either (a) approve the ID, in which case all specimen-level data would automatically be added to the specimen-level database, (b) mark the form as “pending approval” and use custom portals to communicate with the contributor to request more information, additional photographs of species specific characters, and/or request a loan of the specimen, or (c) reject the contribution.

This working space could automatically track activities of all participants and editors, such that a monthly or annual accounting of an individual’s contribution to the system would be generated. In this way, the system could facilitate overcoming part of the credit disconnect noted above (e.g., McDade et al. 2011).

2. A specimen-level database would accumulate all specimen-level data approved by the editors. Fortunately, free open source MySQL specimen-level databases already exist and could be co-opted for this purpose (e.g., Specify6). Specimen-level databases could be directly linked to a ScratchPad or Life Desk for working groups of systematists, as well as Global Biodiversity Informatics Facility (GBIF), National Biological Information Infrastructure (NBII), National Digital Biological Collections Resource, MorphBank, and the Tree of Life Web Project (TOL) and Encyclopedia of Life (EOL) should they expand to include specimen-level data.

3. Perhaps the most exciting part of the system, especially from the perspective of the editors, might be the taxon-specific products that could be generated from the specimen-level data as part of the system. Much like GenBank facilitates organizing and viewing genetic information based on users, interests and queries of genetic data, this system could include algorithms that would allow participants to benefit in useful and unexpected ways from the fruits of their labor. Examples of such products are species distribution maps, analyses of morphological traits over geographic space, lists and keys to regional species, as well as many others. Validated specimen-level locality data is crucial for systematists involved in species distribution modeling and climate change research and these data form the foundation for science-based conservation planning. Historical data from museum collections would add a temporal component, and past species distribution modeling could be compared with present day distributions and incorporated into predictions of change under various future climate scenarios.

While most of the technology needed for this concept is already in place, there is not a present-day example of a portable system that integrates these components into a product that would simultaneously facilitate specimen-level acquisition, train students and parataxonomists, and provide taxon-specific tools to the same community (and other user communities) that drives the system. This new cyber research environment would broaden the community of contributors of biodiversity data without compromising data quality, optimize the efficiency of expert taxonomists, and track the contributions of all participants. It would exponentially increase the amount of validated specimen-level data freely available to create an unlimited number of taxonomic and regional products. It would also use the Internet to facilitate new ways for professional systematists to train future taxonomists.

**Figure 1.**
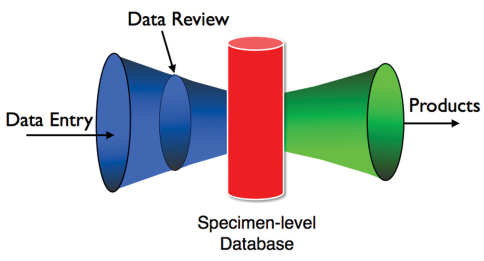
Schematic of integration of components. The blue funnel (on left) depicts web portals and cyberinfrastructure that allows for a diversified contributor pool and speeds the pace of acquiring validated specimen-level data. The data review process provides a peer-review filter to incoming data that helps to ensure data quality. The red column (center) depicts a specimen-level database that stores entries accepted by the editors. This specimen-level database could be directly linked to large-scale initiatives such as GBIF, NBII, National Digital Biological Collections Resource, the Encyclopedia of Life and the Tree of Life, GenBank and MorphBank. The green funnel (on right) represents taxon-specific tools that could be developed utilizing such a rich data set

## Limitations and Challenges

The system as described here would be largely limited to (1) taxa that can be identified by a series of photographs of specific characters, and (2) groups for which there are a critical number of trained experts who are able and willing to serve as editors for such a system.

In addition, most insect collections do not include unique specimen identification numbers associated with each specimen, which provides the necessary link between the physical specimens and any digital information associated with it.

In order for this system to work there would need to be committed team of technical experts, systematists, and software developers to design a seamless data environment and implement the network services to provide the needed interactions within the major processing environments.

## Benefits

If such a system were to be established, many end-users would benefit. First, systematists would benefit from the mobility of vast amounts of specimen-level data. If you are a systematist, imagine if there were such a database filled with specimen records (including photos and georeferences) of your group of interest as you plan your next taxonomic revision. Students, ecologists, and museum scientists would have submitted these specimen records, including images and georeferences, allowing you to generate species distribution maps and easily determine which specimens you would like to request on loan and from where. Broadening the community of contributors would greatly speed the pace of your revisionary work. Students of systematics would benefit from the connections they would make with experts located around the world to efficiently learn the morphological characteristics of their study group.

This system would also help facilitate science-informed conservation planning. One exciting downstream product of such a system is exemplified by a conservation effort in Madagascar. Nowhere is the need for a highly focused effort to conserve species and habitat more apparent. This island nation ranks near the top of virtually every conservation priority list due to its extraordinary levels of endemism and high rates of deforestation, especially within the last 50 years. The Réseau de la Biodiversité de Madagascar (REBIOMA) project plays a critical role in identifying target areas for conservation. The goals of the REBIOMA Project are to attain validated, up-to-date biodiversity data from scientists and to make that information available for conservation planning in Madagascar, and to provide tools to conservation planners and managers to identify conservation priorities for the region. REBIOMA does not provide tools to facilitate the acquisition of these data, but rather relies on systematists to submit these data independently. However, it does provide a means to analyze patterns of diversity and endemism among Malagasy invertebrate (e.g., Fisher and Girman 2000, Paulian 1961, 1972), vertebrate (e.g., Goodman and Ganzhorn 2004, Raxworthy and Nussbaum 1996, 1997) and plant lineages. This information is then used to help define target areas for conservation (Kremen et al. 2008).

The largest bottleneck in the process is the relatively small amount of validated specimen-level data that enters the system in a way that is easily accessible by end-users whether they are professional systematists, biologists, or conservation planners. For these reasons data on megadiverse groups, such as terrestrial arthropods, are altogether lacking in most conservation efforts, and existing biodiversity datasets are heavily concentrated on only a small fraction of animal and plant diversity, particularly in biologically diverse and poorly known regions, even though the under-represented groups are generally the most informative for many purposes, including conservation planning (Kremen et al. 1993, Underwood and Fisher 2006, Kremen 1994, Kremen et al. 1999, Moritz et al. 2001, Good et al. 2006).

In conclusion, we need to develop cyberinfrastructure to promote the identification of insect specimens in our natural history museums. This will allow associated specimen-level data to be included in revisionary work, large-scale specimen-level initiatives and conservation efforts. For taxa such as many groups of insects, which are megadiverse and have relatively few trained experts able to perform species-level identifications, taxon-focused cyberinfrastructure could minimize the labor required by the few taxonomic experts, maximize their expertise, and offer them incentives and appropriate acknowledgement for their work.

## References

[B1] Anonymous (2000) Systematics Agenda 2000: Charting the Biosphere. New York, USA, Technical Report.

[B2] Blagoderov V Brake I Georgiev T Penev L Roberts D Ryrcroft S Scott B Agosti D Catapano T Smith V (2010) Streamlining taxonomic publication: a working example with Scratchpads and ZooKeys. ZooKeys 50: Special issue: 17-28. 10.3897/zookeys.50.539 PMC3088019 21594114

[B3] Ebach MC (2011) Taxonomy and the DNA Barcoding Enterprise. Zootaxa 2742: 67-68.

[B4] Faith DP (1992) Conservation Evaluation and Phylogenetic Diversity. Biological Conservation 61 (1): 1-10. 10.1016/0006-3207(92)91201-3

[B5] Fisher BL Girman D (2000) Biogeography of ants in eastern Madagascar. In: Lourenço WR, Goodman SM (Eds) *Diversity and Endemism in Madagascar*. Mémoires de la Société de Biogéographie, Paris, 331-344.

[B6] Good TC Zjhra ML Kremen C (2006) Dealing with data deficiency in classifying extinction risk: A case study of a radiation of Bignoniaceae from Madagascar. Conservation Biology 20: 1099-1110. 10.1111/j.1523-1739.2006.00473.x 16922226

[B7] Goodman SM Ganzhorn JU (2004) Biogeography of lemurs in the humid forests of Madagascar: the role of elevational distribution and rivers. Journal of Biogeography 31 (1): 47-55. 10.1111/j.1365-2699.2004.00953.x

[B8] Hopkins GW Freckleton RP (2002) Declines in the numbers of amateur and professional taxonomists: implications for conservation. Animal Conservation 5: 245-249. 10.1017/S1367943002002299

[B9] Krell FT (2004) Parataxonomy vs. taxonomy in biodiversity studies – pitfalls and applicability of ‘morphospecies’ sorting. Biodiversity and Conservation 13: 795-812. 10.1023/B:BIOC.0000011727.53780.63

[B10] Kremen C (1994) Biological inventory using target taxa: a case study of the butterflies of Madagascar. Ecological Applications 4: 407-422. 10.2307/1941946

[B11] Kremen C Cameron A Moilanen A Phillips SJ Thomas CD Beentje H Dransfield J Fisher BL Glaw F Good TC Harper GJ Hijmans RJ Lees DC Louis Jr. E Nussbaum RA Raxworthy CJ Razafimpahanana A Schatz GE Vences M Vieites DR Wright PC Zjhra ML (2008) Aligning Conservation Priorities Across Taxa in Madagascar with High-Resolution Planning Tools. Science 320 (5873): 222-226. 10.1126/science.1155193 18403708

[B12] Kremen C Colwell RK Erwin TL Murphy DD Noss RF Sanjayan MA (1993) Terrestrial arthropod assemblages: their use in conservation planning. Conservation Biology 7: 796-808. 10.1046/j.1523-1739.1993.740796.x

[B13] Kremen C Razafimahatratra V Guillery RP Rakotomalala J Weiss A Ratsisompatrarivo J (1999) Designing the Masoala National Park in Madagascar using biological and socioeconomic data. Conservation Biology 13: 1055-1068. 10.1046/j.1523-1739.1999.98374.x

[B14] Maddison DR Schulz KS Maddison WP (2007) The Tree of Life Web Project. Zootaxa 1668: 19-40.

[B15] McDade LA Maddison DR Guralnick R. Piwowar HA Jameson ML Hill A Helgen KM Herendeen PS Vis ML (2011) Biologists needs a modern assessment system for professional productivity. Bioscience 61 (8): 619-625. 10.1525/bio.2011.61.8.8

[B16] Moritz C Richardson KS Ferrier A Monteith GB Stanisic J Williams SE Whiffin T. (2001) Biogeographical concordance and efficiency of taxon indicators for establishing conservation priority in a tropical rainforest biota. Proceedings of the Royal Society of London 268: 1875-1881. 10.1098/rspb.2001.1713 PMC1088822 11564342

[B17] Oliver I Beattie AJ (1993) A Possible Method for the Rapid Assessment of Biodiversity. Conservation Biology 7: 562-568. 10.1046/j.1523-1739.1993.07030562.x

[B18] Oliver I Beattie AJ (1996) Designing a cost-effective invertebrate survey: a test of methods for rapid assessment of biodiversity. Ecological Applications 6: 594-607. 10.2307/2269394

[B19] Paulian R (1961) La Zoogéographie de Madagascar et des Iles Voisines. Faune de Madagascar. 13: 1-442.

[B20] Paulian R (1972) Some ecological and biogeographic problems of the entomofauna of Madagascar. In Battistini R, Richard-Vindard G (Eds) Biogeography and Ecology in Madagascar. W. Junk. The Hague, 411–426.

[B21] Penev L Erwin T Thompson FC Sues HD Engel M Agosti D Pyle R Ivie M Assmann T Henry T Miller J Ananjeva N Casale A Lourenço W Golovatch S Fagerholm HP Taiti S Alonso-Zarazaga M van Nieukerken E (2008) ZooKeys, unlocking Earth’s incredible biodiversity and building a sustainable bridge into the public domain: From “print-based” to “web-based” taxonomy, systematics, and natural history. ZooKeys 1: 1-7.

[B22] Raxworthy, CJ Nussbaum RA (1996) Patterns of endemism for terrestrial vertebrates in eastern Madagascar. In Biogéographie de Madagascar (ed. by W.R. Lourenço), pp. 369-383. ORSTOM, Paris.

[B23] Raxworthy, CJ Nussbaum RA (1997) Biogeographic patterns of reptiles in eastern Madagascar. In Goodman SM, Patterson DB (Eds) Natural change and human impact in Madagascar. Smithsonian Institution, Washington, D.C., 124–141.

[B24] Smith VS Rycroft SD Harman TK,ScottB Roberts D (2009)Scratchpads: a data-publishing framework to build, share and manage information on the diversity of life *BMC Bioinformatics* 2009, **10** (Suppl 14)**:** S6. 10.1186/1471-2105-10-S14-S6 PMC2775152 19900302

[B25] Song H Buhay JE Whiting MF Crandall KA (2008) Many species in one: DNA barcoding overestimates the number of species when nuclear mitochondrial pseudogenes are coamplified. Proceedings of the National Academy of Sciences 105 (36): 13486-13491. 10.1073/pnas.0803076105 PMC2527351 18757756

[B26] Stockle MY Hebert PDN (2008) Barcode of life. Scientific American 299: 82-88. 10.1038/scientificamerican1008-82 18847089

[B27] Underwood EC Fisher BL (2006) The role of ants in conservation monitoring: If, when, and how. Biological Conservation 132: 166-182. 10.1016/j.biocon.2006.03.022

[B28] Will KW Mishler BD Wheeler QD (2005) The perils of DNA barcoding and the need for integrative taxonomy. Systematic Biology 54 (5): 844-851. 10.1080/10635150500354878 16243769

[B29] Wilson EO (2003) The encyclopedia of life. Trends in Ecology & Evolution 18 (2): 77-80. 10.1016/S0169-5347(02)00040-X

